# A New Artificial Neural Network Approach in Solving Inverse Kinematics of Robotic Arm (Denso VP6242)

**DOI:** 10.1155/2016/5720163

**Published:** 2016-08-17

**Authors:** Ahmed R. J. Almusawi, L. Canan Dülger, Sadettin Kapucu

**Affiliations:** ^1^Mechanical Engineering Department, University of Gaziantep, Gaziantep, Turkey; ^2^Mechatronics Engineering Department, University of Baghdad, Baghdad, Iraq

## Abstract

This paper presents a novel inverse kinematics solution for robotic arm based on artificial neural network (ANN) architecture. The motion of robotic arm is controlled by the kinematics of ANN. A new artificial neural network approach for inverse kinematics is proposed. The novelty of the proposed ANN is the inclusion of the feedback of current joint angles configuration of robotic arm as well as the desired position and orientation in the input pattern of neural network, while the traditional ANN has only the desired position and orientation of the end effector in the input pattern of neural network. In this paper, a six DOF Denso robotic arm with a gripper is controlled by ANN. The comprehensive experimental results proved the applicability and the efficiency of the proposed approach in robotic motion control. The inclusion of current configuration of joint angles in ANN significantly increased the accuracy of ANN estimation of the joint angles output. The new controller design has advantages over the existing techniques for minimizing the position error in unconventional tasks and increasing the accuracy of ANN in estimation of robot's joint angles.

## 1. Introduction

Artificial intelligence has become the most modern technology of robotic control. It has many advantages in performance such as precise control and less computing time, in addition to overcoming some mathematical problems in motion and path generation. The main problem of motion control in robotic arm is to find the accurate and reliable solution for inverse kinematics. The calculation of inverse kinematics is necessary in real-time control; the solving of inverse kinematics is computationally complex and requires a very long processing time. Most applications of motion in robotic manipulation require Cartesian space motion control. In inverse kinematics, desired position and orientation of the end effector in Cartesian space are given while the set of robot's joint angles in joint space is calculated.

In general, the solutions of inverse kinematics of a robotic manipulator are geometric, iterative, analytic, or algebraic approaches. Recently, a high focus has been applied on artificial intelligence based methods for inverse kinematics problem solution of general purpose robot. Many studies were done on the implementation of artificial intelligence on a robotic arm to overcome the singular configuration problem of robotic arm. The inverse kinematics of three DOF robotic arm was solved by multilayer network inversion method; the joint angles were estimated for given end effector position in a simulation of three-link robotic arm. The results showed an approximation solution for inverse kinematics [1]. Singularities and uncertainties in arm configurations are the main complications in the kinematics of robot control, in order to have a realistic solution based on one of the heuristic methods; artificial neural network (ANN) was suggested for a nonsurgical robot. The main idea of this approach was the use of ANN to learn the robot system characteristics rather than having to specify an explicit robot system model [2]. A neural network and genetic algorithms were used together to solve the inverse kinematics problem of the nonsurgical robotic manipulator to minimize the error at the end effector and improve the precision of the inverse kinematics solution [3]. The inverse kinematic of redundant manipulators was presented by neural networks (NNs) to obtain the joint angles of the robot using the Cartesian coordinate of the end effector. Position errors of end effector and feasibility of the joint angles were obtained [4]. An artificial neural network was used for controlling 3 DOF robotic manipulator. The methods introduced a nonlinear relation between Cartesian and joint coordinates using multilayer perceptron in artificial neural network. A simulation test was implemented [5]. A neural network architecture was introduced to solve the inverse kinematics problem for robotics manipulators with two degrees of freedom. The neural networks were multilayered perceptron (MLP) with a backpropagation training algorithm for reducing the complexity of the algorithm and calculation (matrix inversion) of inverse geometric of robotic arm. The result showed a mean squared error (MSE) in performance near to 10^−5^ [6]. A high-order-logic theorem was used for solving the kinematics analysis of six-axis revolute joint robot. The approach required an enormous amount of user intervention to overcome the limitation of kinematic analysis [7]. The inverse kinematics problem of the 6 DOF robot is solved by using curved-surface scanning to carry the ultrasonic testing task. Many results for the joint angles were acquired; the method of the shortest distance was assumed to solve the inverse problem of the robot system. A 3D application software was introduced to simulate the motion of ultrasonic trajectory and path planning [8]. The kinematics and singularities of an asymmetrical parallel robotic wrist were investigated by using the method of Lagrange multipliers and considering all the mobile components. The designed model was numerically illustrated to show its computation accuracy [9]. A technique for solving the inverse kinematics problem using artificial neural networks was introduced for a PUMA 560 robot. An inverse kinematic solution was studied by training the neural network with the robot's end effector Cartesian coordinates and its corresponding joint configurations. Results showed mean square error (MSE) of 1.2178, the regression value obtained was 0.87527, and the position errors in *x*-, *y*-, and *z*-axis were 4.93%, 7.29%, and 3.73%, respectively [10]. A solution to the inverse kinematics was required for generating desired trajectories in the Cartesian space (2D). A feedforward neural network was used for planar of three-link manipulators. The result showed the best performance at epoch 9 with mean squared error (MSE) of 0.0054387 [11]. The kinematics of three DOF was introduced for the lower limb of the humanoid robot. Decoupled closed-form solution for the position and orientation was the solution of kinematics; the joint sequences were presented by Denavit-Hartenberg (DH) transformation matrices. Swing phase equations were developed to avoid matrix inversion problems [12]. The kinematic parameters on industrial robot were affected by vibrations disturbance. The error in motion was improved by sensors of accelerometer and gyroscope. The motion profile was analyzed for joint; then, the path tracking of welding task was estimated [13]. Human robot kinematics was identified by geometry kinematics approach to map human arm configuration and stiffness controlled index by hand gesture. The human arm stiffness was estimated within robot experiential stability region. A moving task was implemented to test the performance of geometry kinematics approach on Baxter robot simulator [14]. The geometric approach was used to solve the kinematics of the autonomous positioning of a robotic arm. This modeling and analysis approach was tested by using a five DOF arm with a gripper mounted to the* iRobot* mobile platform [15]. Online robot kinematics parameter errors estimation based on inertial measurement unit (IMU) was presented. It obtained the orientation of the manipulator with the orientation of the IMU in real time. This approach incorporated Factored Quaternion Algorithm (FQA) and Kalman Filter (KF) to the orientation of the IMU [16]. An analytical solution of inverse kinematics for a five DOF spatial parallel micromanipulator was presented. A geometrical mode and structural of system were introduced for the microrobot's task [17]. Forward kinematics and inverse kinematics were calculated and simulation was done for joints and link parameters of six-axis robotic arm. Trajectory planning was described for the requisite motion of the manipulator as a time sequence task [18]. Forward and inverse kinematics of a KUKA robotic arm in the application of a simple welding process were introduced. A general DH representation of forward and inverse matrix was obtained. A movement flow planning was designed and developed for the programming of the robot [19]. The mobile robot with arm (KUKA youBot) and the solving of inverse kinematics problem were introduced. The robot was presented as 8 DOF. The kinematics redundancy of the holonomic platform was presented. Including redundancy parameters, the inverse kinematics solution was suggested [20]. The end effector position and orientation error of a space robot were studied. A geometric parameter identification method was presented based on a laser ranger attached to the end effector. The independence of the geometric parameters was analyzed. Identification equations were derived by simulation which was implemented for different types of robot configuration [21].

This paper introduces a novel solving method for six-axis manipulator robot based on ANN to be used in motion control. It is clear from previous survey that no study has included current joint angles of robot in their ANN. In this study, the ANN architecture has included current joint angles of robot in the input pattern and it improves the performance of proposed ANN in solving inverse kinematics. It is the first ANN that fulfils the requirement of robot precise motion and reduces joint angles error and outcomes in some aspects of the robot tasks.

This paper is organized as follows.  Section 2 presents the kinematics analysis and the required parameters for motion control followed by an explanation of problem formulation. The proposed artificial neural network is described in Section 3.  Section 4 illustrates the system setup.  Section 5 presents the experimental work and discussion of results. Finally, Section 6 concludes this paper.

## 2. Kinematics Analysis

**Figure 1:**
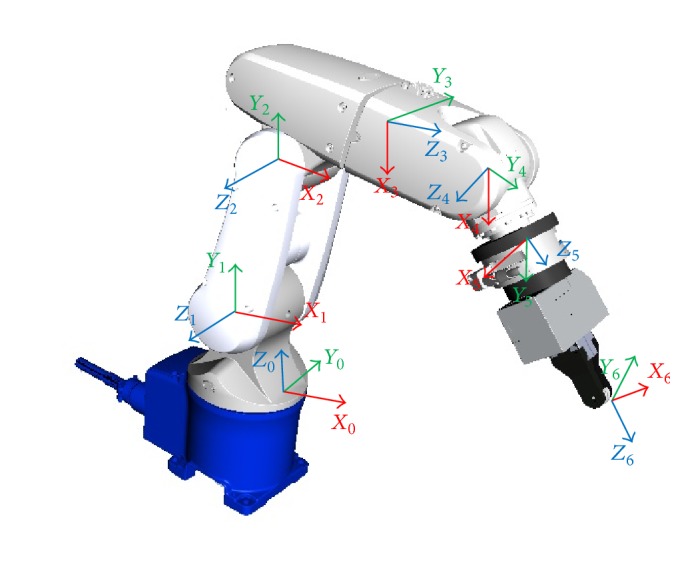
Reference coordinates for the system.

The kinematics of serial manipulator describe the relationship between the joint angles and the position and orientation of its end effector. The kinematics of robot is required in trajectory generation and motion control. The transformation matrices have been used for control. The robotic system is the Denso robot with 6 revolute joints. The kinematics analysis is done after system coordinate frame has been performed, the coordinates *O*_0_, *x*_0_, *y*_0_, *z*_0_ are fixed to the base which is the base frame. The other coordinate frames are attached to the corresponding links. The reference coordinates of the system are shown in Figure 1.

The homogeneous transformation matrix is stated to represent the position and orientation of end effector with respect to base coordinate; a homogeneous transformation matrix *T*_6_^0^ for overall system is as follows:

(1) $$\begin{array}{c} T_{6}^{0}=\left[\begin{array}{cc} R_{6}^{0} & P_{6}^{0}\\ 0 & 1 \end{array}\right], \end{array}$$

where *R*_6_^0^ is a rotation matrix 3 × 3 and *P*_6_^0^ is a position vector of the end effector in the base frame coordinate. The Denavit-Hartenberg DH method is used to analyze the kinematics of Denso robot. The robot transformation matrix has been denoted. The single link homogenous transformation matrix *A*_*i*_ is

(2) Ai=Rotz,θiTransx,diTransx,αiRotx,αi,Ai=Cθi−SθiCαiSθiSαiaiCθiSθiCθiCαi−CθiSαiaiSθi0SαiCαidi0001,

where *i* is link number, *Sθ*_*i*_ = sin *θ*_*i*_, *Cθ*_*i*_ = cos *θ*_*i*_, *θ*_*i*_ is the joint rotation angle, *a*_*i*_ is the length of links, *α*_*i*_ is the twist angles, *d*_*i*_ is the link offsets, and *θ* is the joint angles.

The Denavit-Hartenberg HD parameters of the robot are shown in Table 1.

The system has six links and a gripper. The homogeneous transformation matrix is calculated by multiplication of matrices as follows:

(3) $$\begin{array}{c} T_{gripper}^{0}=A_{1}A_{2}{A_{3}A_{4}A_{5}A}_{6}A_{gripper}, \end{array}$$

where

(4) A1=100000−10010.1250001,A2=100.21010000100001,A3=100−.07500100−1000001,A4=100000−10010.210001,A5=100000100−1000001,A6=10000100001.170001.

The transformation matrix of the gripper is *A*_gripper_; the joint angles and gripper transformation matrix are given; then, the transformation matrix *T*_Home_ of home position is calculated:

(5) $$\begin{array}{c} T_{Home}^{\;}=\left[\begin{array}{cccc} -1 & 0 & 0 & .21\\ 0 & 1 & 0 & 0\\ 0 & 0 & -1 & .24\\ 0 & 0 & 0 & 1 \end{array}\right]. \end{array}$$

**Figure 2:**
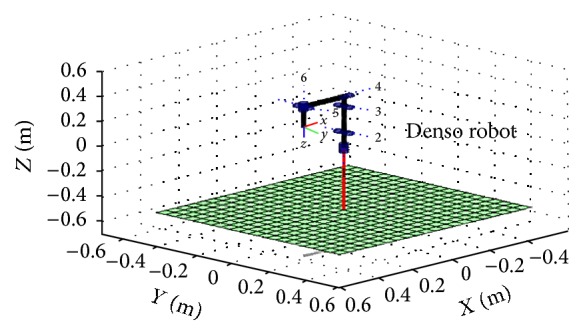
The graphical representation for the robotic system.

The graphical representation of Denso robot is done by MATLAB programming robotics toolbox as shown in Figure 2.

The inverse kinematics might got several solutions produced for each of the joint angles because these are corresponding to many robot configurations such as elbow up, elbow down, wrist up, wrist down, shoulder forward, and shoulder back. The position and orientation of end effector are obtained by forward kinematics as follows:

(6) $$\begin{array}{c} T_{6}^{0}=\text{forward kinematics}\left[\;q\;\right]. \end{array}$$

The joint angles are calculated by inverse kinematic for desired position/orientation of end effector:

(7) $$\begin{array}{c} \left[\;q\;\right]=\text{inverse kinematics}\left(\;T_{6}^{0}\;\right). \end{array}$$

In this study, a new artificial neural network solution for inverse kinematics of (8) is introduced in Section 3:

(8) q1q2q3q4q5q6=inverse  kinematicspxpypzθxθyθz.

### 2.1. The Problem Statement

In general, the desired motion of robot is carried out in the Cartesian coordinate, while the robotic arm motion is controlled by joint coordinate; a solution for the inverse kinematics is very important to be calculated. Solving the inverse kinematics problem for robotic manipulators is a difficult and also quite challenging task. The difficulty of this problem is given by the robot's geometry and the nonlinear trigonometric equations that describe the relationship between the Cartesian space and the joint space. Although a closed-form solution to this problem is preferable in robotics, sometimes it is impossible to find. Therefore, various other ways to determine the solution for inverse kinematics problem were studied such as geometrical solutions and numerical algorithms. This task depends on the designed structure of the robot while many robots such as redundant manipulators do not have an analytical solution for the inverse kinematics.

In this study, two ANNs are designed for inverse kinematics of robotic arm. The first one is the traditional ANN as used in serial robotics inverse kinematics analysis, and the second is the proposed ANN by considering the feedback of current robot configuration (current joint angles) in the design of ANN.

## 3. Traditional Design of Artificial Neural Network

**Figure 3:**
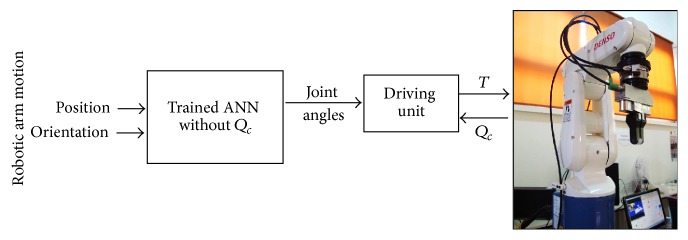
Block diagram of robot motion control by traditional ANN.

A traditional design for ANN is used in many studies [6, 10, 11]. In order to utilize the advantages of this proposed method, traditional ANN is designed in this study to solve the inverse kinematics. In this ANN, the elements in the input layer are six variables, which are the position *P* and orientation *R* of gripper in Cartesian coordinates. The number of hidden layers is ten. The output layer has six elements of the angles of joint *Q*. MATLAB/neural network toolbox is used for training, validation, and testing.  Figure 3 shows a block diagram for traditional ANN and its model as follows:

(9) $$\begin{array}{c} \left[\;Q\;\right]=\text{ANN}\_\text{Traditional}\_\text{Net}\left(\;P,R\;\right). \end{array}$$

The inputs are uniformly enclosed with the workspace of specified position; the corresponding inputs/outputs are computed by solution of forward kinematics. In this way, each position of the robot has a unique joint configuration in the neural network inputs/outputs set.

**Figure 4:**
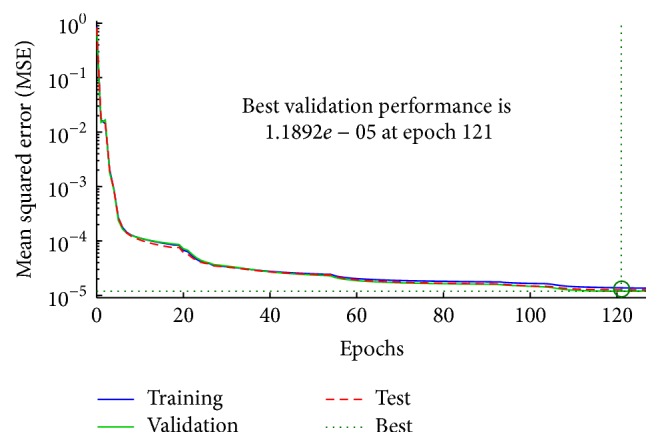
The performance of the traditional neural network.

The training algorithm is the Levenberg-Marquardt backpropagation; it is used to assure fast convergence of the training error and is also a very popular curve-fitting algorithm.  Figure 4 shows the performance of the traditional neural network; the MSE of training is decreased until the validation error is stopped at epoch 121 and MSE was 1.1892*e* ^−5^ in the best performance. A closed relation was between the output and target samples at correlation of 0.99758.

## 4. Proposed Artificial Neural Network Design

Always, robot starts motion from current position, and in most applications robot moves on trajectory on sequential point's path. So the inclusion of current joint configuration in ANN has a positive effect in the estimation of joint angles for the next desired position. In this paper, a novel neural network design is proposed and used to solve the inverse kinematics problem of robotic arm. The proposed method relies on the constraints of the kinematics of robotic arm to achieve robot's motion in an intelligent way with high accuracy in position. The contribution of proposed design is considering the current joint angles of the robot in solving the inverse kinematics of robot by ANN. The inclusion of the current joint angles during training produces a strong network and adjusts the weights with very low error.

**Figure 5:**
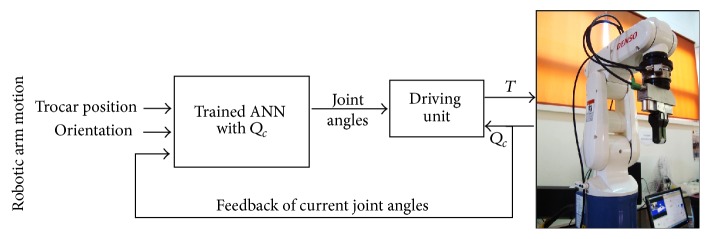
Block diagram of robot motion control by proposed ANN.

The forward kinematics is used to generate the input/target data set which is used in training; the inputs of neural network are the desired position/orientation and current robot joint configuration while the targets are the required joint angles of the robot relative to those points. The proposed neural network has 13 elements in the input layer, which are the gripper position *P* and orientation *R* in Cartesian coordinates and current joint angles *Q*_*c*_ of the robot. The output layer has six elements of joint coordinates, which are the joint angles *Q* of the robot.  Figure 5 shows a block diagram for proposed ANN, the inputs, and outputs of neural network as follows:

(10) Q=ANN_Proposed_NetP,R,Qc,Q=q1,q2,q3,q4,q5,q6,P=x,y,z,R=Rx,Ry,Rz,Qc=q1c,q2c,q3c,q4c,q5c,q6c.

**Figure 6:**
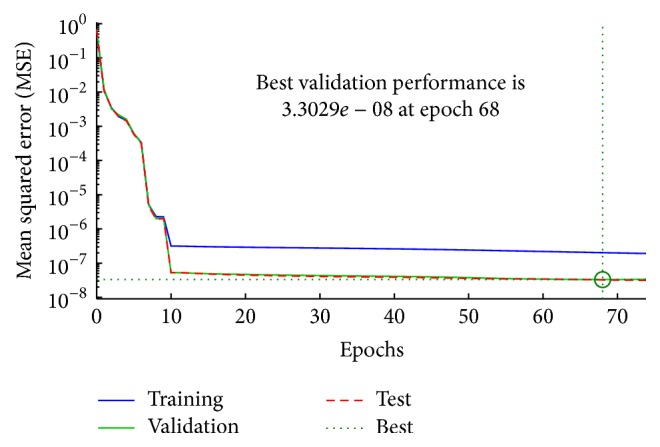
The performance of the proposed neural network.

The performance of the neural network was determined based on the mean squared error (MSE) between the neural network's actual output and the desired output. The performance of the proposed ANN is shown in Figure 6. The differences between the network outputs and target are calculated through the mean squared error (MSE); it drops rapidly through the learning process; the MSE of training is decreased until the validation error is stopped at epoch 68 and MSE was 3.3029*e* ^−8^ in the best performance. A closed relationship was between output and target samples at correlation 0.99999. The new outputs of the network are checked by the test data, the training samples were 2800, and the validated and test samples were 600 for each one.

## 5. System Setup

**Figure 7:**
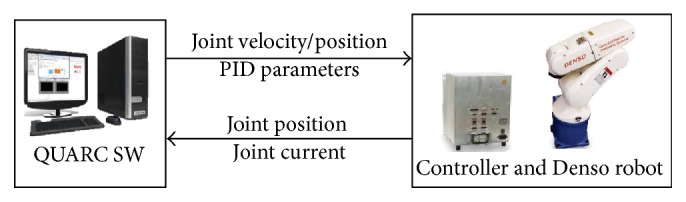
The control parameters between robot and QUARC software.

Denso robot VP6242 is a six-axis industrial robot (Quanser Company). Artificial neural network is implemented on this system for predicting joint angles during real-time Cartesian motion. The robot is communicated with MATLAB/Simulink via TCP/IP. An open source program (QUARC) control software is supported; the QUARC software is executed in Simulink for real-time application. A blockset is used to connect Simulink program with Denso driving unit. The joint positions and joint currents (ampere) are reading on PC while the joint velocities/positions are sending to robot. The joint PID parameters or joint feedforward gains are adjustable and user can deal with them. The total arm length is 420 mm, and the payload is 2.5 kg.  Figure 7 shows the control parameters between robot and QUARC software SW.

**Figure 8:**
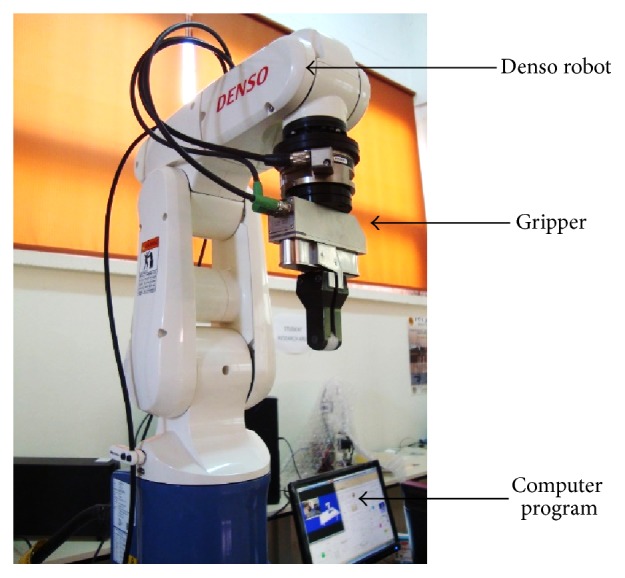
Robotic arm system setup.

The gripping system is an electrical drive gripper WSG 32 SCHUNK; it is a very precise handling system for medium parts weight up to 0.5 kg, and it has integrate high-sensitivity sensor to detect parts in a gripping force of 5–50 N and opening of up to 65 mm. The gripper is required for pick-and-place part or other handling activities.  Figure 8 shows robotic system full setup.

**Figure 9:**
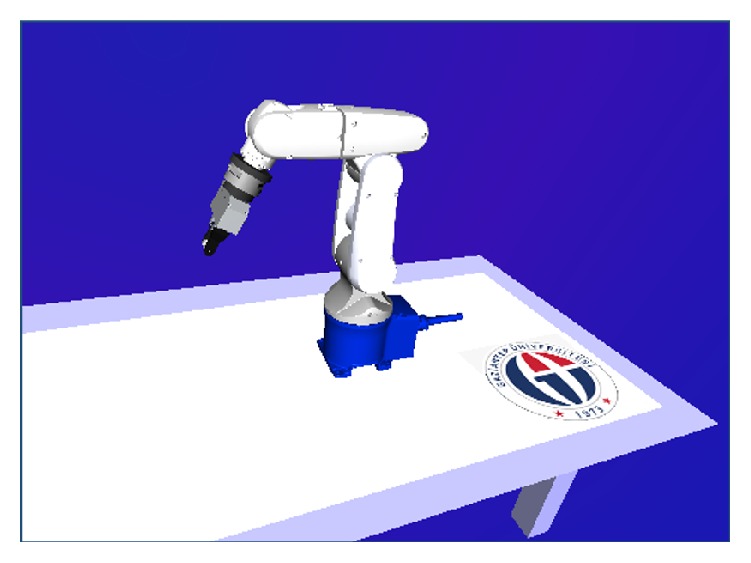
Virtual model of the robotic system.

A virtual model is connected with Simulink to visualize the system by using Simulink 3D animation. The virtual model and the system model are combined to create a virtual robotic system environment. This virtual model can record and present the motion path of real robot during experiments.  Figure 9 shows the virtual model of robotic system.

## 6. Experiment Results and Discussion

**Figure 10:**
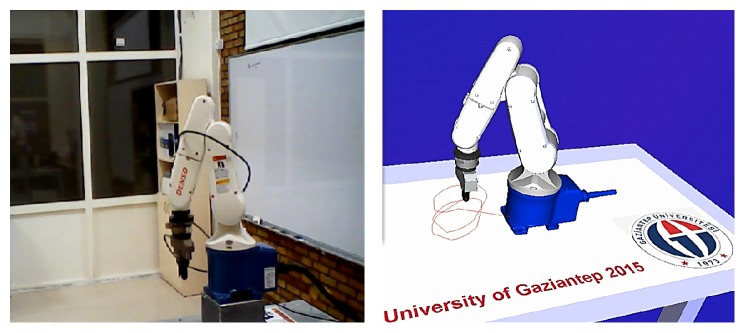
Experiment on the robotic motion demonstration by ANN (movement of end effector through helical path).

After the training of proposed ANN and traditional ANN is completed, the experiments of movement are carried out by the robotic system. The gripper motion is in a helical path within the workspace area. The input path for gripper tip is generated by *P*_*x*_(*k*) = *c*_1_ sin⁡ (*k*), *P*_*y*_(*k*) = *c*_2_ cos (*k*), and *P*_*z*_(*k*) = *c*_3_ *k*. In this experiment, the robot has to follow a sinusoidal rotation in *x*-axis and *y*-axis while the motion in *z*-axis is a linear path; the constants *c*_1_ and *c*_2_ are the amplitude of radius on the *x*- and *y*-axis, respectively; the constant *c*_3_ is the pitch of a helix, and *k* is the sample time.  Figure 10 shows the real robotic system and the virtual model to demonstrate helical motion path by ANN. The inverse kinematics solution for robot motion is achieved by proposed ANN and traditional ANN.

**Figure 11:**
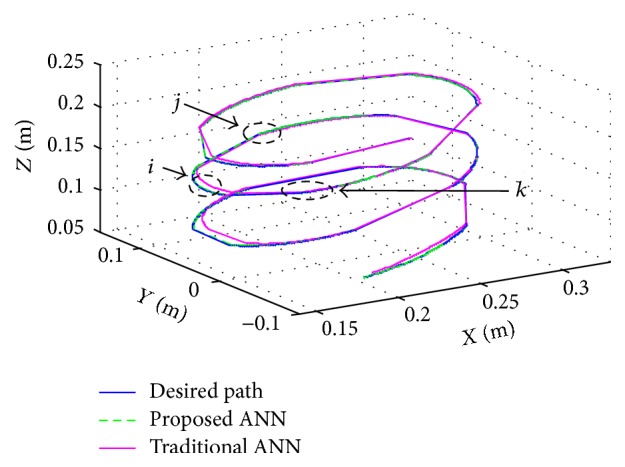
Paths configuration generated by robot in two different ANNs. The selected points *i*, *j*, and *k* are enlarged portions in Figure 12.

The movements of robot are executed in circular and linear motion. The pitch of path of the end effector is changed by varying the position in *z* direction relative to workspace coordinates. The rotation of end effector is performed by changing the position to *x* and *y* directions. Two paths are generated by using proposed ANN and traditional ANN with desired path.  Figure 11 illustrates the paths configuration of the end effector by the robot movement.

**Figure 12:**
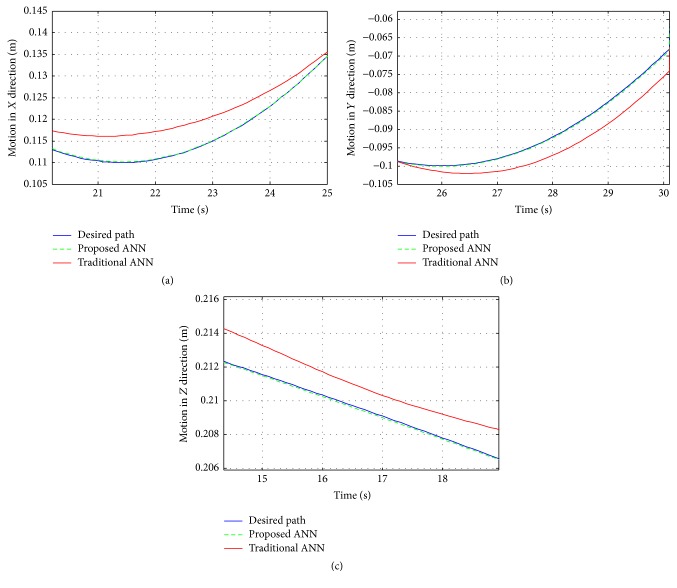
Robot motion by two ANNs: (a) the motion in *x* direction, (b) the motion in *y* direction, and (c) the motion in *z* direction.

The motion of the robot is in a Cartesian space. In Figure 12(a), the motion in *x* direction is an enlargement of the time period (20 s–25 s).  Figure 12(b) is the motion in *y* direction enlarged for the time period (26 s to 30 s).  Figure 12(c) is the motion in *z* direction enlarged for the time period (14 s to 19 s). Figures mentioned above show the differences in *x*, *y*, and *z* directions for motion resulting from proposed ANN and traditional ANN and the high accuracy and precision of proposed ANN.

**Figure 13:**
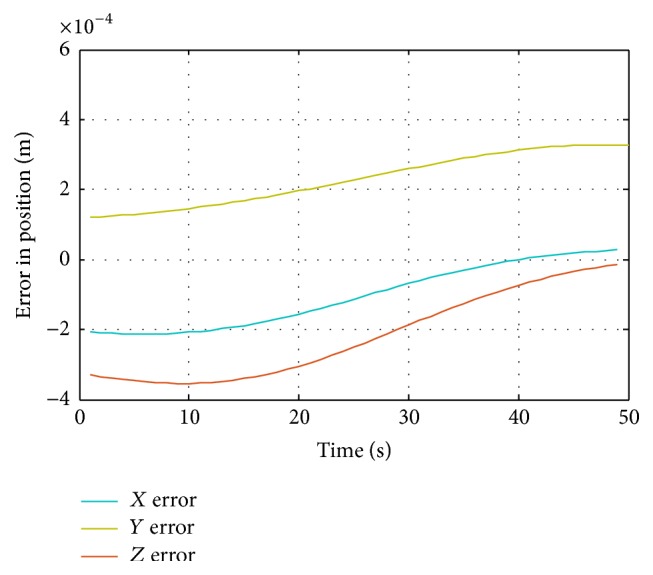
Proposed ANN motion error in *x*, *y*, and *z* direction.

**Figure 14:**
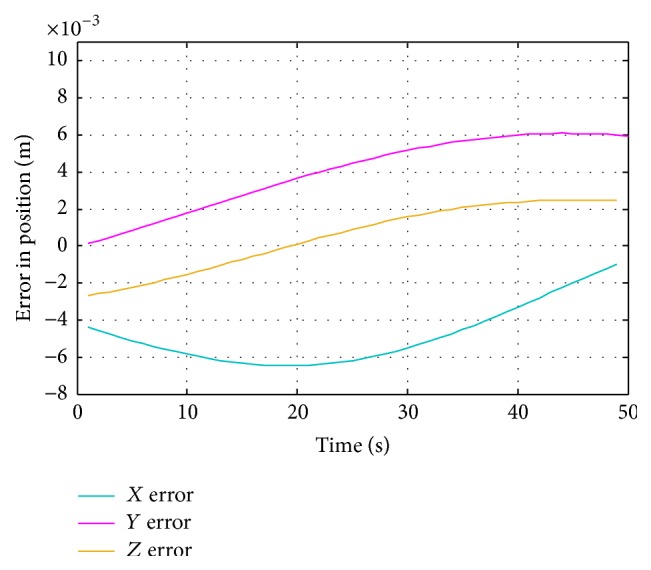
Traditional ANN motion error in *x*, *y*, and *z* direction.

The differences between desired path and artificial neural network path are measured by calculating the error in position from proposed ANN and traditional ANN with respect to position from desired path.  Figure 13 shows the error in *x*, *y*, and *z* position for proposed ANN, while Figure 14 shows the errors in *x*, *y*, and *z* position for traditional ANN.

The motion curves of proposed ANN are much more precise than the motion curves of traditional ANN in estimating the joint angles of the robot for desired positions. The maximum error in *x* direction for proposed design of ANN was near 0.22 millimeters and for traditional design of ANN2 was 6.5 millimeters. The maximum error in *y* direction for proposed design of ANN was less than 0.3 millimeters and for traditional design of ANN2 was near 6 millimeters. The maximum error in *z* direction for proposed design of ANN was 0.35 millimeters and for traditional design of ANN2 was near 2.5 millimeters.  Table 2 shows the error percentages of proposed and traditional ANNs.

According to the ANN based solving inverse kinematics results in the literature [2–6, 10, 11], the proposed approach in this study has a minimized error in the inverse kinematics solution.  Table 3 shows system performance comparison between this study and other studies from literature. The high accuracy and the low MSE that are obtained in this work can be obviously seen.

The errors are increased in some points and are reduced in other points; the point of high error is because of estimating position by ANN while the point of low error is the point that is near the samples of training set in ANN.

The proposed ANN has given higher accuracy and precision in position than the traditional ANN, and this method is applicable in precise robotic motion.

## 7. Conclusions

This study introduced a very accurate solution for inverse kinematics by using the artificial neural network to overcome the drawbacks of traditional ANN controller. A new design of artificial neural network ANN has been proposed for the optimal robot motion control in Cartesian coordinates. In order to evaluate the integral performance of the system, the current joint angles information was added to the traditional ANN based inverse kinematics solution. The proposed design showed improvement in performance of end effector in some aspects.

The motion of robot has been executed; it satisfies the constraint of robotic arm motion by the designed structure. Robot kinematics are analyzed, and position/orientation of end effector in different configurations are studied.

A comparison has been carried out between two ANNs. The parameters of motion and the errors were calculated. The results showed that the proposed ANN has superior performance in terms of the joint angles estimation. The design of ANN compared to other techniques is applicable for some of the most difficult and challenging problems of kinematics. These results have proved the effectiveness of the proposed ANN. The inclusion of current configuration of joint angles in ANN increased the accuracy of ANN estimation and succeeded in mapping between input position and joint angles output.

## Supplementary Material

This is an experiment on robotic system motion control by using artificial neural network, the motion is applied in a helical path within the workspace area.

## Figures and Tables

**Table 1:** DH parameters of the Denso robot.

Link *i*	*θ*_*i*_	*d*_*i*_	*a*_*i*_	*α*_*i*_
1	*q*_1_	0.125	0	pi/2
2	*q*_2_	0	0.21	0
3	*q*_3_	0	−0.075	−pi/2
4	*q*_4_	0.21	0	pi/2
5	*q*_5_	0	0	−pi/2
6	*q*_6_	0.07	0	0

**Table 2:** Performance of robotic system by ANNs.

Parameters	Proposed ANN	Traditional ANN
*P*_*x*_ error%	0.17	5.78
*P*_*y*_ error%	0.36	7.25
*P*_*z*_ error%	0.12	1.28
MSE	3.3029*e* ^−8^	1.1892*e* ^−5^
Regression	0.99999	0.99758

**Table 3:** System performance comparison between this study and other studies mentioned in the literature.

Study	System	DOF	Method	MSE	Hidden layers	Errors
Proposed	Denso	6	ANN	3.3*e* ^−8^	10	*x* = 0.17% *y* = 0.36% *z* = 0.12%

Luv et al., 2014 [10]	PUMA 560	6	ANN	1.217	30	*x* = 6.42% *y* = 4.90% *z* = 2.92%

Hasan et al., 2010 [2]	FANUC M-710i robot	6	ANN	~1	1	*x* = 3.34% *y* = 6.72% *z* = 0.35%

Toshani and Farrokhi, 2014 [4]	Simulation PA-10 robot	7	NNs with optimization	1	—	End effector 5 mm

Duka, 2014 [11]	Planar simulation	3	NNT	0.0054	1	—

Köker, 2013 [3]	Stanford	6	ANN	2.38	25	End effector 4.28 mm

Nanda et al., 2012 [5]	Simulation	3	ANN, FLANN	>1	20	—

Daya et al., 2010 [6]	simulation	2	NNT	5.24*e* ^−5^	2	—

## References

[1] Ogawa T., Kanada H. (2010). Solution for Ill-posed inverse kinematics of robot arm by network inversion. *Journal of Robotics*.

[2] Hasan A. T., Ismail N., Hamouda A. M. S., Aris I., Marhaban M. H., Al-Assadi H. M. A. A. (2010). Artificial neural network-based kinematics Jacobian solution for serial manipulator passing through singular configurations. *Advances in Engineering Software*.

[3] Köker R. (2013). A genetic algorithm approach to a neural-network-based inverse kinematics solution of robotic manipulators based on error minimization. *Information Sciences*.

[4] Toshani H., Farrokhi M. (2014). Real-time inverse kinematics of redundant manipulators using neural networks and quadratic programming: a Lyapunov-based approach. *Robotics and Autonomous Systems*.

[5] Nanda S. K., Panda S., Subudhi P. R., Das R. K. (2012). A novel application of artificial neural network for the solution of inverse kinematics controls of robotic manipulators. *International Journal of Intelligent Systems and Applications*.

[6] Daya B., Khawandi S., Akoum M. (2010). Applying neural network architecture for inverse kinematics problem in robotics. *Journal of Software Engineering & Applications*.

[7] Wu A., Shi Z., Li Y. (2015). Formal kinematic analysis of a general 6R manipulator using the screw theory. *Mathematical Problems in Engineering*.

[8] Lu Z., Xu C., Pan Q., Zhao X., Li X. (2015). Inverse kinematic analysis and evaluation of a robot for nondestructive testing application. *Journal of Robotics*.

[9] Wu G. (2014). Kinematics and dynamics of an asymmetrical parallel robotic wrist. *Journal of Robotics*.

[10] Luv A., Kush A., Ruth J. (2014). Use of artificial neural networks for the development of an inverse kinematic solution and visual identification of singularity zone(s). *Procedia CIRP*.

[11] Duka A. V. (2014). Neural network based inverse kinematics solution for trajectory tracking of a robotic arm. *Procedia Technology*.

[12] Said A., Rodriguez-Leal E., Soto R., Gordillo J. L., Garrido L. (2015). Decoupled closed-form solution for humanoid lower limb kinematics. *Mathematical Problems in Engineering*.

[13] Elvira-Ortiz D. A., de Jesus Romero-Troncoso R., Jaen-Cuellar A. Y., Morales-Velazquez L., Osornio-Rios R. A. (2016). Vibration suppression for improving the estimation of kinematic parameters on industrial robots. *Shock and Vibration*.

[14] Liang P., Ge L., Liu Y., Zhao L., Li R., Wang K. (2016). An augmented discrete-time approach for human-robot collaboration. *Discrete Dynamics in Nature and Society*.

[15] Clothier K. E., Shang Y. (2010). A geometric approach for robotic arm kinematics with hardware design, electrical design, and implementation. *Journal of Robotics*.

[16] Du G., Zhang P. (2013). IMU-based online kinematic calibration of robot manipulator. *The Scientific World Journal*.

[17] Prusak D., Kobus K., Karpiel G. (2014). Kinematics analysis of a novel five-degree-of-freedom spatial parallel micromanipulator. *Journal of Robotics*.

[18] Kishore K., Srinath A., Jugalanvesh G., Premsai R., suresh M. (2013). Kinematic analysis and simulation of 6Dof KukaKr5 robot for welding application. *International Journal of Engineering Research and Applications*.

[19] Dahari M., Tan J.-D. Forward and inverse kinematics model for robotic welding process using KR-16KS KUKA robot.

[20] Sharma S., Kraetzschmar G. K., Scheurer C., Bischoff R. Unified closed form inverse kinematics for the KUKA youBot.

[21] Liu Y., Jiang Z., Liu H., Xu W. (2012). Geometric parameter identification of a 6-DOF space robot using a laser-ranger. *Journal of Robotics*.

